# Measuring, and identifying predictors of, women's perceptions of three types of breast cancer risk: population risk, absolute risk and comparative risk

**DOI:** 10.1038/sj.bjc.6604910

**Published:** 2009-02-10

**Authors:** C Apicella, S J Peacock, L Andrews, K Tucker, M B Daly, J L Hopper

**Affiliations:** 1 Department of Public Health, Centre for Molecular, Environmental, Genetic and Analytic Epidemiology, Level 1, 723 Swanston Street, Carlton, VICTORIA, 3053, Australia; 2 Centre for Health Economics in Cancer, British Columbia Cancer Agency, 675 West 10th Avenue, Vancouver, British Columbia, V5Z 1L3, Canada; 3 School of Population and Public Health, University of British Columbia, 5804 Fairview Avenue, Vancouver, British Columbia, V6T 1Z3, Canada; 4 Hereditary Cancer Clinic, Department of Medical Oncology, Prince of Wales Hospital, High St, Randwick, New South Wales, 2031, Australia; 5 Department of Population Science, Fox Chase Cancer Center, 333 Cottman Avenue, Philadelphia, PA, 19111-2497, USA

**Keywords:** Ashkenazi, breast cancer, perceived risk, genetic counselling, *BRCA1*, *BRCA2*

## Abstract

Although a key function of cancer genetics services is to provide risk information, to date there has been little consistency in the way in which breast cancer risk perception has been measured. The aims of the study were to measure estimates of (i) population risk, (ii) absolute risk and (iii) comparative risk of developing breast cancer for Ashkenazi Jewish women, and to determine predictors of breast cancer risk perception. Of 152 women, 107 (70%) completed all questions. The mean (s.d.) estimates for population risk, absolute risk and comparative risk were 22.7% (15.9), 31.8% (20.6) and 1.9-fold (1.9), respectively. Most women overestimated population risk. Women at population risk generally overestimated the population risk and their own absolute risk, yet understood they are at the same risk as the population. Those with a family history understood that they are at increased risk, but underestimated the extent to which their familial risk is increased. Anxiety, high estimation of population risk and lesser family history predicted overestimation of absolute risk, whereas high estimation of population risk and a strong family history predicted underestimation of comparative risk.

A key function of cancer genetics services is to provide clients with cancer and genetic risk information, for the purpose of informing clients, aiding decision-making, reducing anxiety and possibly improving adherence to appropriate screening practices. In the context of breast cancer, this includes discussion of both the woman's risk of developing breast cancer and of the major factors contributing to this risk, in particular her family cancer history. These services may further try to identify clients’ misconceptions about breast cancer risk and modify their beliefs so as to provide clients with a more realistic view of their own breast cancer risk.

An accurate and reliable measure of women's breast cancer perception is required to assess whether women accurately estimate their breast cancer risk before genetic counselling, and if not, to assess to what extent cancer genetics services are successful in modifying inaccurate breast cancer risk perceptions. However, after more than a decade of literature, there is little consensus on how risk perception should be measured.

It has previously been recognised that risk perception measurement may depend on both the statistical measure and the population studied (Dupont and Plummer, 1996). Risk perception has been measured both as an absolute risk and as a comparative risk, and using a range of reference times, so there are a variety of ways of presenting risk. A common way of measuring absolute risk has been to ask: ‘what do you think is your chance of getting breast cancer’. A common way of measuring comparative risk has been to ask: ‘what do you think is your chance of getting breast cancer compared to…’, with comparison usually made to women in the general population either specified to be of the same age as the individual, or with no age specification. Time frames that have been used for these questions include ‘in the next 5 years’, ‘in the next 10 years’, ‘to age 70’, ‘to age 80’ or ‘in your lifetime’. Responses have been presented in categories (e.g., ‘high’, ‘moderate’ or ‘low’) or as continuous variables, usually as ratios or percentages (Watson *et al*, 1999; Hopwood *et al*, 2003; Schapira *et al*, 2004; Gurmankin Levy *et al*, 2006; Zajac *et al*, 2006).

With regard to the population studied, if estimates are pooled for women of differing actual risk categories (e.g., across the spectrum of family histories), important issues may be masked. This is because risk perception and its determinants may differ by actual risk. More recent publications have compared, rather than pooled, women from different absolute risk categories or limited their study to women from a specific absolute risk category (Hopwood *et al*, 2003; van Dooren *et al*, 2004; Haas *et al*, 2005; Edwards *et al*, 2006).

Another concept not often studied in the context of risk perception for breast cancer is that of an anchor point. There is an extensive literature examining perceptions of risk in the context of transportation safety and environmental hazards (Lichtenstein *et al*, 1978; Slovic 1987; Hakes and Viscusi, 2004; Andersson and Lundborg, 2007). This literature provides significant evidence to support the notion that people determine their risk of a given event by using a reference (anchor) point and they better estimate their absolute risk of a given event if they are first provided with an accurate reference risk. This suggests that people may think well in terms of comparative risk and it may be possible to alter absolute risk perceptions by including counselling about the reference risk. To our knowledge, the study by Dillard *et al* (2006) is the only study to date that has examined anchor points in the context of breast cancer risk perception.

In this study, we asked Ashkenazi Jewish women participating in a study of the breast cancer susceptibility genes *BRCA1* and *BRCA2* their perceptions of: (i) the population risk of breast cancer, (ii) their absolute risk of breast cancer and (iii) their comparative risk of breast cancer to determine predictors of breast cancer risk perception as a function of women's family histories and their actual absolute and comparative risks.

## Materials and methods

The study was approved by the Human Research Ethics Committees of the Peter MacCallum Cancer Institute (Melbourne), The Prince of Wales Hospital (Sydney) and The University of Melbourne. Written informed consent was obtained from all participants.

### Subjects

Participants of the Australian Jewish Breast Cancer Study (AJBCS) were self-identified as being of Ashkenazi Jewish descent, living in Melbourne or Sydney, and reported having (a) had an earlier diagnosis of breast or ovarian cancer themselves or (b) a first- or second-degree male or female relative who had been diagnosed with breast or ovarian cancer (Apicella *et al*, 2006). They were recruited through announcements in the media (local Jewish newspapers (32%) and other newspapers (3%)), approaches including brochures to general practitioners, gynaecologists, breast surgeons and oncologists (19%), the family cancer genetics clinics associated with this study (9%), information evenings (11%) and friends or relatives (26%).

Eligible subjects for this study were participants of the AJBCS without breast cancer or prophylactic bilateral mastectomy; with complete personal characteristics and family cancer history data; and who returned a follow-up questionnaire with all risk questions completed.

### Baseline data collection

At recruitment into the AJBCS, demographic data and information on personal and family cancer history were collected. Blood samples were taken and genetic testing performed for the mutations 185delAG and 5382insC in *BRCA1* and 6174delT in *BRCA2* (Apicella *et al*, 2006).

### Follow-up questionnaire

A follow-up questionnaire was mailed to all AJBCS participants on an average of 44 months (7–60 months) after their enrolment. For women who learnt their genetic test result, an average of 39 months (5–57 months) had elapsed since their attendance at a cancer family genetics clinic. The follow-up questionnaire included a genetic knowledge instrument consisting of nine true–false questions. This instrument was developed specifically for knowledge about breast cancer and the *BRCA1* and *BRCA2* genes (Hughes *et al*, 1997; Lerman *et al*, 1997). Participants were also asked about recent life events, including whether a close relative had recently died, or was recently diagnosed with cancer. The State Trait Anxiety Inventory (STAI) Y2 version was administered (Spielberger, 1983).

### Questions on risk perception

The follow-up questionnaire included questions on risk perception that were developed for this study. These questions asked participants to (1) Provide a numeric value for their perception of population risk; *‘*What percentage (number from 0 to 100) of women in the population do you think will get breast cancer in their lifetime?*’* (2) Choose an option to describe their comparative risk. ‘Do you think YOUR chance of getting breast cancer is higher or lower than this, or about the same? Please tick the box to indicate your response’ (options: no risk; much lower; a little lower; same; a little higher; much higher; definitely will get breast cancer) (3) Provide a numeric value for their perception of their own absolute risk of developing breast cancer. ‘In your opinion, what percentage (number from 0 to 100) reflects your chance of getting breast cancer in the future?’

The participants’ numeric value for their perception of their comparative risk was then derived by dividing their perception of their absolute risk by their perception of the population risk. For example, a woman who responded that the population risk was 25%, and her absolute risk was 50%, would have a numeric perceived comparative risk of 50/25=2.0.

### Genetic information and risk counselling

Participants received a genetic information session at the time of enrolment. Those who wished to receive their genetic test result also attended a cancer family genetics clinic to receive further genetic counselling and later attended a genetics clinic for a result disclosure appointment. The genetic information sessions were conducted by the researchers and the clinic appointments were conducted by 14 genetic counsellors and clinic geneticists, based at six clinics. Risk counselling was provided during the genetic information session and at each of the clinic appointments, so that all participants in this study received risk counselling on at least one occasion and most received it on at least two or three occasions.

The risk counselling provided to participants included discussion of the population risk of getting breast cancer and of the participant's absolute and comparative risk of developing breast cancer. The NBCC guide for health professionals on familial aspects of breast and ovarian cancer is used by Australian breast cancer family genetics clinics. Information on their category and the estimated absolute and comparative risk ranges were provided to participants at both the genetic information session and the genetic counselling appointment(s) using the *guide* (National Breast Cancer Centre, 2006). That is, women were counselled using this *guide* by showing them the categories and explaining which risk category they belonged to and the associated absolute and comparative risk range. In addition to the *guide*, some genetic counsellors also presented breast cancer risks in other ways. The extent, content or techniques used by counsellors for additional risk counselling were not recorded.

### Actual risk and coding the participants’ risk estimates

For this study, we used the risk figures provided by the researchers and genetic counsellors to the participants as estimates of the actual risk. For population risk, participants were told the figure of a 9% lifetime risk, and so responses within a range of 7–12% were coded as ‘correct’. Participants who estimated the population risk to be above 12% were coded as having overestimated, and those selecting less than 7% were coded as having underestimated.

Given that all participants were counselled with estimates of their absolute risk and comparative risk of developing breast cancer determined from the NBCC *guide*, we used the categories of the *guide* as an estimate of the participant's actual risk. The family history categories are: Family History Category 1, 2 and 3. Individuals in Family History Category 1 are those at population risk of developing breast cancer, and women in this category were informed that they were at the same risk as the general population, which is 9%. Responses in the range of 7–12% were therefore coded as correct (as for the estimate of the population risk), corresponding to a comparative risk of 0.8- to 1.3-fold. Participants in Family History Category 2 and 3 are those at estimated risks (as per NBCC *guide*) of 13–25% and 26–49%, respectively, corresponding to comparative risks of 1.4- to 2.8-fold and 2.9- to 5.4-fold, respectively. Participants’ estimates of their own absolute risk, and the calculated comparative risk, were coded as having being estimated correctly if they were within the stated ranges; overestimated if above the upper limit of those ranges; and underestimated if below the lower limit of those ranges.

In addition, a number of women in this study learnt that they carry an ancestral mutation in *BRCA1* or *BRCA2*. These women were provided with different risk information from those who did not carry these mutations. Available data on the penetrance of these mutations changed over the course of this study, and so women who received their results in 1996 or early in 1997 were counselled that a mutation conferred an 85–90% risk of developing breast cancer, whereas those who received their result later were counselled that this conferred a risk of ‘about 50%’. For mutation carriers, we therefore coded any responses for absolute risk between 50 and 90% and comparative risk between 5.5- and 10-fold as having been estimated correctly; those above these ranges were coded as having overestimated; and those below these ranges were coded as having underestimated.

For the question asking women to select a response that best describes their comparative risk in words, women in any family history category who selected ‘a little lower’ or ‘much lower’ were coded as underestimated.

Those in Family History Category 1 who selected ‘same’ were coded as correctly estimated, and those who selected ‘a little higher’ or ‘much higher’ were coded as overestimated.

Those in Family History Category 2 who selected ‘same’ were coded as underestimated, those who selected ‘a little higher’ as correctly estimated and those who selected ‘much higher’ were coded as overestimated.

Those in either Family History Category 3 or the Mutation Carrier Category who selected ‘same’ or ‘a little higher’ were coded as underestimated, and those who selected ‘much higher’ were coded as correctly estimated. For these two categories, a woman was only coded as overestimated if she selected ‘definitely will get breast cancer’.

### Statistical analysis

Regression analysis was performed to identify predictors of women's risk estimates. Five analyses were conducted with the dependent variables on the log odds of women's estimates of: (i) population risk, (ii) absolute risk, (iii) comparative risk, (iv) overestimation of absolute risk and (v) underestimation of comparative risk. Independent variables were indicators of respondents’ personal characteristics. Analyses were preformed using Stata v8.2.

## Results

Of the 339 AJBCS participants who were sent the follow-up questionnaire (average 44 months after enrolment in the study), 256 (76%) returned them. However, 100 women with breast cancer and 4 unaffected women who had had prophylactic bilateral mastectomy were excluded. Of the remaining 152 women eligible for this study, 109 (72%), 144 (95%) and 128 (84%) gave a response for the numeric estimate of population risk of breast cancer, their own absolute risk of developing breast cancer and their comparative risk of developing breast cancer, respectively. Complete personal characteristic data, family cancer history data and risk question data were available for 107 participants (70%).

Table 1 describes the personal characteristics of 107 full respondents. The majority were aged between 25 and 54 years, and were parous. About half held a university degree and the mean genetic knowledge score was 7.1 (1.5). Older women had more children (*P*<0.05), whereas younger women were more likely to have a university degree (*P*<0.01) and had better genetic knowledge (*P*<0.05). The mean STAI score was 37.7 (1.5). Recent cancer diagnosis in the family was associated with being a carrier of an ancestral mutation (*P*<0.01).

Table 1 further shows that 95 of the 107 full respondents attended a genetics clinic appointment and that 88 received their genetic test result. That is, all participants received the risk counselling at least once (as part of the genetic information session delivered at enrolment); 95 women received it on at least two separate occasions (the genetic information session and genetic clinic appointment); and 88 women received it on three separate occasions (the genetic information session, genetic clinic appointment and genetic clinic result disclosure appointment).

Table 2 shows participants’ responses to the risk questions. The mean (s.d.) estimate for the population risk was 22.7% (15.9) and was similar across all family history categories. More than half overestimated population risk (estimations >12%), whereas only 5% underestimated (estimations <6%). The mean (s.d.) estimate for their own absolute risk was 31.8% (20.6) and increased with family history, ranging from 22.7% for those in Family History Category 1 to 45% for mutation carriers. Overall, about half overestimated absolute risk. The mean (s.d.) estimate for comparative risk was 1.9-fold (1.9) and increased with family history, ranging from 1.2-fold for those in Family History Category 1 to 3.0-fold for mutation carriers. Almost half of all women underestimated their comparative risk of developing breast cancer, whereas the three-fourth of women at actual comparative risk >2.8 (i.e., Family History Category 3 and mutation carriers) underestimated their comparative risk.

From Table 2, it can be seen that overall the proportion who underestimated their comparative risk in words was 46% compared with 48% who underestimated the numeric comparative risk. Similarly, 40 (65%) above population risk underestimated their comparative risk in words and 40 (65%) above population risk underestimated their numeric comparative risk.

Multivariate regression was performed on the log odds of participants’ estimates of population risk, their own absolute risk and their calculated comparative risk (their estimate of their own absolute risk divided by their estimate of the comparative risk). Results are reported in Table 3 for the women who provided complete responses for all risk questions, and for whom all personal characteristic data were available.

Genetic knowledge and anxiety scores were significantly associated with estimated population risk. An increase in genetic knowledge score was significantly associated with a decrease in the estimate of the population risk, whereas an increase in anxiety score was significantly associated with an increase in estimate of the population risk.

Genetic knowledge score was significantly associated with an increase in the estimated absolute risk. Further, having a strong family cancer history (Family History Category 3) and being a mutation carrier (Family History Category Carrier) were also significantly associated with an increase in the estimated absolute risk. There were no statistically significant independent associations between genetic knowledge scores and family history.

Family history and mutation carrier status were significantly associated with increasing estimates of comparative risk. That is, having a moderate (Family History Category 2) or strong (Family History Category 3) family cancer history and being a mutation carrier (Family History Category Carrier) were associated with higher estimates of their comparative risk compared with women at population risk (Family History Category 1).

Finally, regression analysis was used to identify predictors of overestimation of absolute risk and predictors of underestimation of comparative risk (see Table 4). Those with a recent death in the family were less likely to overestimate their absolute risk, whereas higher anxiety scores were associated with greater overestimation. A higher estimate of the population risk and a lower family history category were also associated with a greater overestimation of absolute risk.

The only variables significantly associated with underestimation of comparative risk were the estimates of population risk and the family history category variables. Higher estimates of population risk and higher family history category (i.e., higher actual comparative risk) were associated with greater underestimation of comparative risk.

## Discussion

In this study, we used a novel approach to determine Ashkenazi Jewish women's estimates of their risk of developing breast cancer. Women were asked their numeric estimates of the population risk and their own absolute risk of developing breast cancer. Their responses were used to calculate their numeric estimate of their comparative risk of developing breast cancer. Further, a comparative risk question, asking the women to choose a category that describes their comparative risk in words, was included to determine whether the numeric values provided by the women reflected their understanding.

We have shown that the response in words selected by these women to describe their comparative risk of developing breast cancer was remarkably consistent with the numeric comparative risk calculated by dividing their percentage estimate of the population risk with their percentage estimate of their own absolute risk.

We have further shown that women's estimates of the population risk were far higher than the actual population risk of 9%. Overall, participants estimated that more than one in five Australian women will develop breast cancer, when in reality less than half that number will be diagnosed with the disease in their lifetime. Although higher anxiety scores and lower genetic knowledge scores were associated with higher estimates of population risk, the variables included in regression analysis accounted for only a small amount of variation in the data. That is, the number selected for the population risk was for the most part random. This finding is consistent with the economics and psychology literature on risk perception, and suggests that the population risk estimate may be an anchor point upon which other estimates are made (Slovic *et al*, 1981).

For most women, their estimated absolute risk was far higher than their actual absolute risk. The regression model for absolute risk showed that the absolute risk estimate was significantly associated with family history category. This suggests that, in general, women in this study have understood that their family history is associated with an increased risk of developing breast cancer.

The regression model for comparative risk showed that increasing family history category was associated with a higher estimate. This is further evidence that these women understood that a family history is associated with their own risk of developing the disease. In contrast, the regression of underestimation of comparative risk showed that there was greater underestimation with increasing family history category. This, in combination with the finding that 79% of women in Family History Category 3 and 89% of mutation carriers underestimated their comparative risk, suggests that women may not have understood the extent to which their family history of breast cancer increased their risk of disease.

This could, however, also have been due to a ‘ceiling effect’. For women in the highest family history categories to have obtained a correct comparative risk, they would need to have estimated extremely high values for their own absolute risk (near 100% or greater), given their high estimate for the population risk. There is a possibility, therefore, that the underestimation of comparative risk in these women could be because they have reached a ceiling or upper limit.

The economics of safety literature provides some insight into this issue. Data have been collected where one half of the study participants were asked to estimate the risk of three events (e.g., death in a car crash, motorcycle crash and bus crash), and the other half were provided with the actual risk figure for the first event (death in a car crash) and asked to estimate the risk of the second and third events (death in a motorcycle and bus crash). This study showed that in the first instance all three scenarios were highly overestimated, but once an anchor point had been placed for the first, the estimate of the second and third scenario came much closer to the actual absolute risk and also much closer to the actual comparative risk (Philips *et al*, 1989).

In the context of women in the higher family history categories, therefore, it would be of interest to ask their estimates of their absolute risk when the question is repeated after provision of the actual population risk. If the absolute risk estimate does not alter much (as it is usually correct in the higher family history categories), then the data in this study showed a ceiling effect, and women have in fact correctly understood the degree to which their family history increases their risk. However, if the women provide lower estimates of their absolute risk, then they have not understood the extent to which their family history increases their own risk.

For women in the lowest family history category (those at population risk), the estimate of their absolute risk was generally far too high, but once their estimate of the population risk (also far too high) was taken into account, their comparative risk estimate was generally correct. That is, these women have placed their anchor point too high but it seems that they understood that they are at the same risk as other women in the general population.

Participants in the study were drawn from a cohort of Ashkenazi Jewish women participating in a study of the *BRCA1* and *BRCA2* genes. It is not known to what extent these findings may be applicable to other groups. The principles and questions used in this study could easily be applied to other populations to determine whether findings in this study apply to those groups.

## Conclusion

In this study of Ashkenazi Jewish women we have shown that population risk is substantially overestimated. Although significant predictors were found, we were unable to explain most of the variation in the population risk estimate, and we suggest that it may be simply an anchor point upon which women base their other risk estimates. We also found that absolute risk estimates are substantially overestimated by women at population risk, but that this overestimation decreases with increasing family history. Related to this, estimated comparative risk was correct for about half of women at population risk, but substantial underestimation occurs with increasing family history.

These findings suggest that women at population risk generally understand their risk, and may be able to provide a better estimate of their absolute risk with better education about the population risk. These findings also suggest that women with a stronger family history understand that their risk is higher due to their family cancer history, but may not understand the full extent to which their risk is higher due to this risk factor. However, the possibility that the underestimation of comparative risk in these women was due to a ceiling effect should be examined in further studies.

## Figures and Tables

**Table 1:** Descriptive table

**Personal characteristics**	***N***	**Percent**
*Age (*N=*107)*		
25–54 years	74	69
55–74 years	32	30
75 years +	1	1
Mean (s.d.)	49.4 (10.3)	
		
*Number of children (*N=*102)*		
0	21	21
1	9	9
2 or 3	65	64
4 or more	7	7
Mean (s.d.)	1.9	(1.7)
		
*University degree (*N=*107)*		
Yes	62	42
No	45	58
		
*Actual risk of developing breast cancer (*N=*106)*		
Family History Category 1 (AR 7–12%)	43	41
Family History Category 2 (AR 13–25%)	29	27
Family History Category 3 (AR 26–49%)	25	24
Carrier of ancestral mutation (AR 50–90%)	9	8
		
*Genetic knowledge (max 9) (*N=*107)*		
1–3 (poor)	3	3
4–6 (fair)	25	23
7–9 (good)	79	74
Mean (s.d.)	7.1 (1.5)	
		
*Anxiety (STAI Trait) (*N=*104)*		
<40	64	62
40–54	33	32
55–70	7	7
Mean (s.d.)	37.7 (1.5)	
		
*Recent death of a relative (*N=*105)*		
Yes	21	20
No	84	80
		
*Recent cancer of a relative (*N=*103)*		
Yes	22	21
No	81	79
		
*Attended genetic clinic (*N=*107)*		
Yes	95	89
No	12	11
		
*Received genetic test result (*N=*107)*		
Yes	88	82
No	19	18

AR=absolute risk.

**Table 2:** RR, absolute and population risk by family history

	**Family History Category 1; RR 0.8–1.3; AR 7–12% (*n*=40)**	**Family History Category 2; RR 1.4–2.8; AR 13–25% (*n*=29)**	**Family History Category 3; RR 2.9–5.4; AR 26–49% (*n*=24)**	**Mutation carriers; RR 5.5–10.0; AR 50–90% (*n*=9)**	**Total (*N*=102)**
*Population risk*					
Mean estimate (s.d.)	21.3 (16.4)	24.1 (15.4)	25 (16.8)	18.4 (13.6)	22.7 (15.9)
					
Overestimating	18 (45)	19 (66)	15 (63)	4 (44)	56 (55)
Correct	21 (53)	9 (31)	6 (25)	5 (56)	41 (40)
Underestimating	1 (3)	1 (3)	3 (13)	0 (0)	5 (5)
					
*Absolute risk*					
Mean estimate (s.d.)	22.7 (18.1)	31.8 (16.9)	42 (21.4)	45 (23.2)	31.8 (20.6)
					
Overestimating	22 (55)	16 (55)	12 (50)	0 (0)	50 (49)
Correct	11 (28)	10 (34)	5 (21)	6 (67)	32 (31)
Underestimating	7 (18)	3 (10)	7 (29)	3 (33)	20 (20)
					
*Calculated comparative risk (estimated own absolute risk/estimated population risk)*					
Mean estimate (s.d.)	1.2 (0.8)	1.6 (0.9)	2.9 (3.2)	3.0 (1.8)	1.9 (1.9)
					
Overestimating	8 (20)	5 (17)	4 (17)	0 (0)	17 (17)
Correct	23 (58)	9 (31)	3 (13)	1(11)	36 (35)
Underestimating	9 (23)	15 (52)	17 (71)	8 (89)	49 (48)
					
*Comparative risk in words*					
Overestimating	6 (15)	1 (3)	0 (0)^*^	0 (0)^*^	7 (7)
Correct	27 (68)	11 (38)	6 (25)	4 (44)	48 (47)
Underestimating	7 (18)	17 (59)	18 (75)	5 (66)	47 (46)

AR=absolute risk; RR=relative risk.

**Table 3:** Predictors of population risk, absolute risk and RR

	**Log odds of the estimate of population risk**			**Log odds of the estimate of absolute risk**			**Log odds of the estimate of comparative risk**		
	**Number of obs=96**			**Number of obs=94**			**Number of obs=70**		
	***R*^2^=0.28**			***R*^2^=0.52**			***R*^2^=0.74**		
**Independent variables**	**Coefficient^a^**	**95% CI**	***P*-value**	**Coefficient^a^**	**95% CI**	***P*-value**	**Coefficient^a^**	**95% CI**	***P*-value**
Age	−0.02	−0.04–0.00	0.12	0.00	−0.02–0.02	0.87	0.01	−0.03–0.05	0.55
University degree	−0.25	−0.67–0.17	0.25	0.11	−0.28–0.50	0.58	−0.01	−0.71–0.69	0.97
Parity	0.12	−0.03–0.26	0.12	0.04	−0.10–0.18	0.55	−0.01	−0.25–0.24	0.95
Genetic knowledge score	−0.23	−0.34 to −0.11	0.00	0.15	0.02–0.27	0.02	−0.09	−0.30–0.12	0.38
Recent cancer diagnosis in a relative	−0.09	−0.55–0.38	0.71	0.01	−0.41–0.44	0.96	0.06	−0.69–0.82	0.86
Recent death of a relative	−0.17	−0.65–0.31	0.49	0.13	−0.57–0.30	0.54	0.40	−0.45–1.25	0.36
Anxiety	0.02	0.00–0.04	0.02	0.00	−0.02–0.02	0.99	−0.02	−0.05–0.02	0.32
Oophorectomy	0.51	−0.03–1.05	0.07	0.00	−0.51–0.50	0.99	0.07	−0.78–0.91	0.88
Received gene test result	−0.17	−0.78–0.45	0.59	−0.10	−0.66–0.45	0.71	0.50	−0.47–1.48	0.31
Family History Category 2	0.31	−0.15–0.77	0.19	0.36	−0.06–0.78	0.09	2.98	2.19–3.79	0.00
Family History Category 3	0.30	−0.19–0.79	0.23	0.74	0.29–1.19	0.00	3.96	3.13–4.80	0.00
Mutation carrier	0.02	−0.67–0.72	0.95	1.18	0.52–1.85	0.00	4.29	3.19–5.39	0.00
Estimate of population risk	—	—	—	—	—	—	0.00	−0.02–0.02	0.95
Constant	−0.06	1.85–1.72	0.94	−3.27	−5.08 to −1.46	0.00	−3.22	−6.32–0.12	0.04

CI=confidence interval; RR=relative risk.

a Unstandardised *β*-coefficient.

**Table 4:** Predictors of overestimation of absolute risk and underestimation of RR

	**Log odds of the estimate of overestimation of absolute risk**			**Log odds of the estimate of underestimation of comparative risk**		
	**Number of obs=49**			**Number of obs=56**		
	***R*^2^=0.74**			***R*^2^=0.70**		
**Independent variables**	**Coefficient^a^**	**95% CI**	***P*-value**	**Coefficient^a^**	**95% CI**	***P*-value**
Age	−0.01	−0.02–0.00	0.15	−0.00	−0.02–0.02	0.85
University degree	−0.08	−0.27–0.11	0.38	0.09	−0.28–0.46	0.62
Parity	0.01	−0.07–0.09	0.75	0.00	−0.12–0.12	0.98
Genetic knowledge score	0.05	−0.01–0.12	0.11	−0.04	−0.14–0.07	0.48
Recent cancer diagnosis in a relative	0.22	−0.05–0.48	0.11	0.03	−0.33–0.40	0.86
Recent death of a relative	−0.28	−0.51 to −0.05	0.02	0.05	−0.35–0.45	0.81
Anxiety	0.01	0.00–0.03	0.03	−0.01	−0.02–0.01	0.43
Oophorectomy	0.24	−0.06–0.53	0.11	0.13	−0.28–0.54	0.53
Received gene test result	−0.15	−0.43–0.13	0.27	−0.42	−1.02–0.18	0.16
Family History Category 2	−0.37	−0.59 to -0.15	0.00	0.67	0.14–1.21	0.02
Family History Category 3	−0.65	−0.91 to −0.39	0.00	1.57	1.04–2.10	0.00
Mutation carrier	−1.16	−1.91 to −0.42	0.00	2.01	1.41–2.62	0.00
Estimate of population risk	0.01	0.01–0.02	0.00	0.01	0.00–0.03	0.02
Constant	0.35	−0.62–1.32	0.47	−0.42	−1.96–1.13	0.59

CI=confidence interval; RR=relative risk.

a Unstandardised *β-*coefficient.
